# Supplementary material for: MetaRibo-Seq measures translation in microbiomes
Source: Nat Commun. 2020 Jun 29;11:3268. doi: 10.1038/s41467-020-17081-z (PMC7324362; doi:10.1038/s41467-020-17081-z)
Supplement: Supplementary file 10 — Supplementary Data 7 [file 41467_2020_17081_MOESM10_ESM.zip › File2/Confidence_High_Taxonomy/402337_out.krona.html]

Javascript must be enabled to view this page.

members
magnitude
magnitudeUnassigned
count
unassigned
taxon
rank

402337\_out

14

14
superkingdom
2

14
1239
phylum

class
186801
14

14
order
186802

186803
family
14

841
genus
14

2049040
species

SRS011084\_contig\_number\_15814
1


SRS014948\_contig\_number\_21748SRS015431\_contig\_number\_43110SRS015960\_contig\_number\_22308SRS050941\_contig\_number\_20694SRS063518\_contig\_number\_21334SRS076756\_contig\_number\_19526SRS098061\_contig\_number\_10567SRS101433\_contig\_number\_contig-100\_2374.89119SRS1055034\_contig\_number\_12969SRS143780\_contig\_number\_28471SRS144362\_contig\_number\_43716SRS148159\_contig\_number\_31758SRS893252\_contig\_number\_7767
species
166486
13
